# Oxygen, life forms, and the evolution of sexes in multicellular eukaryotes

**DOI:** 10.1038/s41437-020-0317-9

**Published:** 2020-05-15

**Authors:** Elvira Hörandl, Franz Hadacek

**Affiliations:** 1 Department of Systematics, Biodiversity and Evolution of Plants, University of Goettingen, Göttingen, Germany; 2 Department of Plant Biochemistry, University of Goettingen, Göttingen, Germany

**Keywords:** Evolutionary theory, Biosynthesis

## Abstract

The evolutionary advantage of different sexual systems in multicellular eukaryotes is still not well understood, because the differentiation into male and female individuals halves offspring production compared with asexuality. Here we propose that various physiological adaptations to oxidative stress could have forged sessility versus motility, and consequently the evolution of sexual systems in multicellular animals, plants, and fungi. Photosynthesis causes substantial amounts of oxidative stress in photoautotrophic plants and, likewise, oxidative chemistry of polymer breakdown, cellulose and lignin, for saprotrophic fungi. In both cases, its extent precludes motility, an additional source of oxidative stress. Sessile life form and the lack of neuronal systems, however, limit options for mate recognition and adult sexual selection, resulting in inefficient mate-searching systems. Hence, sessility requires that all individuals can produce offspring, which is achieved by hermaphroditism in plants and/or by multiple mating types in fungi. In animals, motility requires neuronal systems, and muscle activity, both of which are highly sensitive to oxidative damage. As a consequence, motility has evolved in animals as heterotrophic organisms that (1) are not photosynthetically active, and (2) are not primary decomposers. Adaptations to motility provide prerequisites for an active mating behavior and efficient mate-searching systems. These benefits compensate for the “cost of males”, and may explain the early evolution of sex chromosomes in metazoans. We conclude that different sexual systems evolved under the indirect physiological constraints of lifestyles.

## Introduction

Understanding why and how eukaryotic sex evolved remains one of the key unresolved questions in evolutionary biology. Most evidently, sex is intrinsically costly (Birdsell and Wills 2003, Otto 2009): first, meiosis bears the risk of breaking up favorable gene combinations, and genetic recombination is not necessarily a selective advantage (Otto 2009); second, sexual reproduction requires two parents to produce offspring. The costs of outcrossing additionally include the need of mate searching and finding, together with the risk of being exposed to predators during these activities. If only one parent (the female) is capable of producing offspring, as is the case in most animals, then an asexual female produces twice as much progeny as a sexual one (“cost of males”; Smith and Maynard-Smith 1978). Many hypotheses exist that attempt to explain the paradox of sex (Birdsell and Wills 2003), but none of them have so far received unequivocal and general reception for all eukaryotes (West et al. 1999, Neiman et al. 2017).

A subgroup of these theories regards oxidative damage of DNA and mutagenesis as major forces for the evolution of sex in eukaryotes. The rise of oxygen concentrations in the Earth’s atmosphere and oceans was one of the most important events in life history, and is thought to have triggered the origin of eukaryotic life. Oxygen tolerance could have represented a pivotal factor in shaping eukaryote evolution because it required basic adaptations in cell structure, organization, and metabolism (Dowling and Simmons 2009; Gross and Bhattacharya 2010; Speijer et al. 2015; Hörandl and Speijer 2018). Eukaryotic meiosis–mixis cycles may have evolved as a homologous recombinational tool that repairs endogenous oxidative DNA damage of nuclear DNA (Michod 1995; Hörandl and Hadacek 2013; Bernstein and Bernstein 2013; Speijer 2016). Sex and recombination also eliminate deleterious mutations efficiently by natural selection against mutants. By contrast, asexual lineages can suffer from irreversible accumulation of deleterious mutations (Muller’s ratchet; Muller 1964; Kondrashov 1988).

While DNA restoration hypotheses can explain the benefit of meiosis–mixis cycles, the origin of sexes or the evolutionary advantages gained by this trait remain unaccounted for. The last common ancestor of eukaryotes (LECA) was probably unisexual, whereas bi-, tri-, and multisexual systems evolved later in eukaryote kingdoms (Heitman 2015). Bisexual systems are most common and established in many clades, in opisthokonts (comprising animals and fungi), plants, alveolates, and heterokonts. Hence, while meiosis–mixis cycles represent ancestral eukaryotic traits, sexual systems are derived and specific for the particular kingdoms of eukaryotes (Heitman 2015). Animals have mostly separate sexes, with male and female individuals. In contrast to animals, land plants have diverse sexual systems, and among flowering plants, by far the largest group, most species are hermaphrodites and produce male and female gametes on the same individuals; only 5–6% of species have separate sexes (i.e., male and female plants, dioecy; Renner 2014). Fungi evolved a great diversity of asexual and sexual reproductive systems, including unisexual, bisexual, and multisexual systems (Heitman 2015). Combined sexes and self-sex mating (homothallism) is found throughout their kingdom (Lee et al. 2010). The selective forces driving the evolution of different sexual systems have so far remained unclear.

**Fig. 1:**
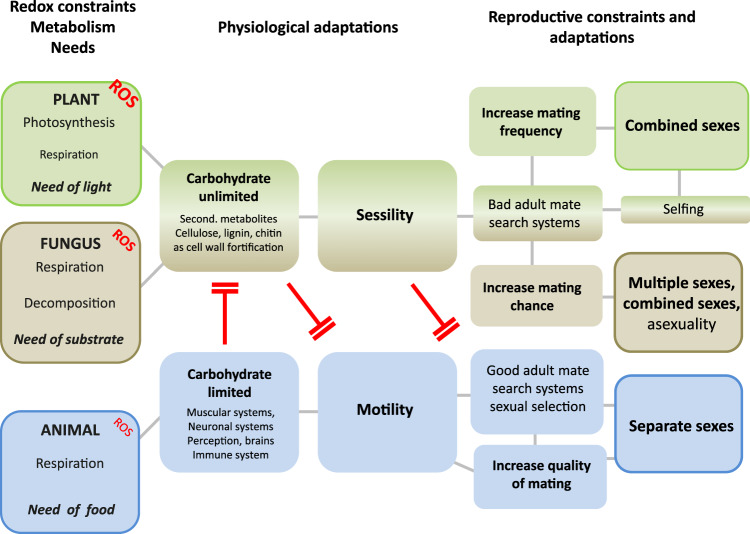
Overview of the main connections of constraints of exposure to oxidative stress, metabolic adaptations, and needs, with differentiation of sexes in plants (green), fungi (brown), and animals (blue). Only predominant features/pathways of kingdoms are shown (exceeding ca. 20% of species, after Renner 2014; Jarne and Auld 2006; Heitman 2015). Lines indicate a positive (fostering) interaction of traits; red T symbols blocking effects.

Here we will propose a link between the physiological constraints of lifestyles and the evolution of different sexual systems. We compare animals, plants, and fungi in terms of potential physiological constraints that can be caused by oxidative stress (Fig. 1). These physiological constraints enforce sessile lifestyles in plants and fungi, and motility in animals. We understand “motility” here as active movement of the whole organism (i.e., excluding passive transport, occupation of space by growth, and movements of just some organs or of certain life stages). We will argue that these physiological adaptations to oxidative stress offer explanations why we find sessile and motile life forms, and how these life forms promote the evolution of two or more sexes in multicellular complex organisms (animals, fungi, and plants). In this context, we will not focus on protists due to lack of cell differentiation, but provide arguments on why we have separate sexes in the great majority of metazoans, but combined sexes in most plants.

We present here an outline of the sections:

1. We will revisit fundamental theories that oxidative stress directly shaped the evolution of different gametes (anisogamy and oogamy).
2. We will propose a hypothetical scenario in which adaptations to combined oxidative stress caused by photosynthesis and respiration shaped the lifestyle and sexual systems of land plants.
3. We will present the hypothesis that similar constraints exist in saprotrophic, symbiotic, or parasitic sessile fungi, and how their lifestyles favored the evolution of either combined or multiple sexes.
4. We will review physiological constraints in motile heterotrophic animals, namely oxidative stress arising from muscle activity, and the sensitivity of the neuronal system to oxidative damage. We will argue that motility favored the evolution of separate sexes.
5. We conclude that trade-offs exist for balancing redox homeodynamics and lifestyle. Eukaryotic organisms may combine just two major sources of oxidative stress (photosynthesis plus respiration, or decomposition plus respiration, or motility plus respiration), but the adaptations for these lifestyles preclude the evolution of traits involving additional oxidative stress. These constraints indirectly shape the evolution of mating systems.

### Uniparental organellar inheritance and the mitochondrial theory of anisogamy

During the establishment of sex in the first unicellular eukaryotes, organelles (mitochondria and plastids) were mostly inherited uniparentally (e.g., Allen and De Paula 2013). The selective forces for this phenomenon might be (1) the avoidance of overreplication, (2) the avoidance of mito-cytoplasmic conflicts after mixing organelles from different individuals (Beekman et al. 2014), (3) the improvement of fitness under mitochondrial mutation accumulation, or (4) selfish conflict (Hadjivasiliou et al. 2013). All these theories do not necessarily exclude each other.

Multicellular eukaryotes separate differentiated somatic cells from germline cells (Bendich 2010). In animals, this happens very early in development, while in plants and fungi, the germline only differentiates in adult organisms. In multicellular eukaryotes, the soma gets adapted to active metabolism and this leads inevitably to oxidative damage, aging, and death. By contrast, the immortal germline adapts to inheritance, and hence remains metabolically quiescent, conserves pristine organelles, and undergoes costly nuclear meiotic repair (Hörandl 2009; Bendich 2010). Hence, we have to differentiate between gametes (i.e., the actually fusing cells and nuclei) and adult organisms that produce them. Accordingly, we will restrict our focus to multicellular, differentiated eukaryotes in the ongoing text. Adults may produce gametes of the same size (isogamy) or of different size (anisogamy, with different resource allocations of small male gametes and bigger female gametes to offspring) (Lewis 1987). Isogamy is mostly found in unicellular eukaryotes, and may evolve into multiple mating types in some multicellular fungi (Constable and Kokko 2018). Anisogamy evolved already early in eukaryotes (Lewis 1987). Many eukaryotes developed a specialized form of anisogamy, i.e., the development of smaller, motile male gametes, and bigger, immotile female gametes (Lewis 1987, Allen 1996). This form is often termed “oogamy” in the literature, but many transitions exist between anisogamy and oogamy (Lewis 1987).

Allen (1996) proposed that two different selective forces shaped the phenomenon of uniparental reproduction and having only one motile gamete type: (1) motility requires highly active mitochondria, which results in oxidative damage of these organelles in male gametes during their competitive race toward egg cells; (2) nonhomologous DNA repair of such oxidative membrane lesions in mitochondria of male gametes, which, however, can be potentially mutagenic. Excluding mutated organelles of the male gamete from the zygote clearly contributes to the fitness of offspring (Hadjivasiliou et al. 2013). Hence, mitochondria of male gametes are not inherited. On the female side, (proto-) mitochondria in egg cells remain largely inactive and undamaged before fertilization; thus, they are maternally inherited without being harmed by oxidative damage and mutagenic DNA repair (de Paula et al. 2013). Likewise, in plants, plastids are inactive in female gametes and predominantly inherited maternally (Bendich 2010). Moreover, male gametes are small and can be produced in huge numbers, which increases the efficiency of selection against harmful mutations (Otto et al. 2015; Immler and Otto 2018). By contrast, female gametes are usually bigger and are produced in smaller amounts, which reduces the efficiency of purifying selection (Hörandl 2009; de Paula et al. 2013). Mathematical modeling of the effects of differential gamete size and of DNA metabolic damage on mortality suggests that disruptive selection acts on gametes (Bonsall 2006). Consequently, variable mortality of gametes due to differential DNA damage favors the evolution of anisogamy (Bonsall 2006). This phenomenon relies on a division-of-labor principle in terms of motility of male gametes versus low risk of oxidative damage to organelles in female, immotile gametes. Anisogamy (including oogamy) is often seen as the driver of different sex roles on the organism level (Lehtonen et al. 2016, Janicke et al. 2016).

However, multicellular eukaryotes may have combined sexes and produce both gamete types (hermaphrodites), or they may produce just one type of gametes and hence have separate sexes (male and female individuals) (Eppley and Jesson 2008). Gamete differentiation cannot explain the different distributions of sexes in adult animals, plants, and fungi (Cavalier-Smith 2010). The following sections will focus on the adult, differentiated organisms and their basic physiological constraints, which we propose to determine their sexual systems.

### Effects of oxidative stress on plants

#### Evolution, physiology, and lifestyle of photosynthetic organisms

Photosynthesis evolved in cyanobacteria about 2.4 billion years ago (Knoll and Nowak 2017), and was integrated into eukaryontic physiology via endosymbiosis. Many observations support the notion that sex evolved in aquatic, unicellular, motile, and unisexual eukaryotes (Heitman 2015). Whether these organisms were auto-, hetero-, or mixotrophic, is unknown, and in protists, there is also no clear correlation to motile/sessile life forms. For instance, dinoflagellates represent photosynthetic, unicellular, mobile organisms. The oldest fossil exhibiting multicellular organization and differentiation of male/female reproductive types, however, is a sessile, photosynthetic organism, namely the red-algae-like *Bangiomorpha pubescens*. This fossil is 1.2 billion years old and points out that sexes emerged quite early in eukaryotic evolution (Butterfield 2000). Eukaryotic life diversified further in aquatic systems until the Ordovicium, when colonization of land started (Knoll and Nowak 2017). Compared with terrestrial habitats, marine, photosynthetic organisms live in a world that requires less buffering against oxidative stress caused by temperature fluctuations. Aquatic environments thus facilitated the evolution of various combinations of more or less motile, heterotrophic or autotrophic lifestyles in multicellular eukaryotes. For instance, some extant multicellular, anisogamous algae, such as *Volvox*, combine photosynthesis with flagellate motility. High- temperature stress triggers formation of reactive oxygen species (ROS) in *Volvox*, which leads to sex-bearing forms (Nedelcu et al. 2004). However, water has a high specific heat capacity, and in oceans, mean daily temperature variations are typically very narrow (<0.3 °C) (Morgan 2016). Nevertheless, despite the stress-buffering capacities of water, no marine photosynthetic organism is known that developed motility using neuromuscular systems.

After the colonization of land in the Ordovician/Silurian (Knoll and Nowak 2017), plants became obligately sessile. Terrestrial life imposes a much higher stress resulting from UV irradiation, drought, higher temperature fluctuations, and eventually soil salinity (de Vries and Archibald 2018). Land plants have bigger, immobile egg cells; mosses, ferns, Cycads, and *Ginkgo* have mobile male gametes, whereas in conifers and flowering plants, even the male gametes are transported passively.

**Fig. 2:**
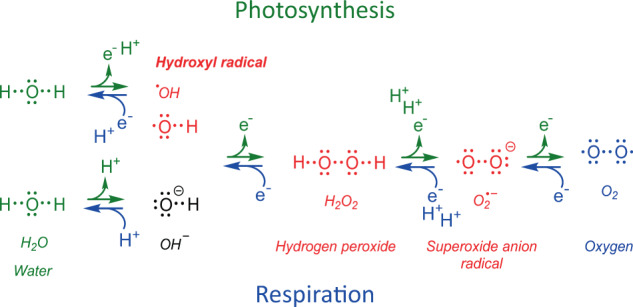
Comparative oxygen chemistry in photosynthesis and aerobic respiration. Substrates, products, and reactive oxygen species (ROS) formation in eukaryotic oxygenic photosynthesis and aerobic respiration; electron donors, green; electron acceptors, blue; ROS, red. Lewis structures aim to illustrate electron transfer dynamics. For anaerobic respiration see Supplementary electronic materials S1.

To understand this complete loss of motility, even of male gametes in conifers and flowering plants, the basic physiological constraints of photosynthesis must be considered. Plants have to cope with two major potential sources of cellular oxidative stress, namely photosynthesis and respiration, especially under high light conditions (Mullineaux et al. 2018). Figure 2 summarizes the basic chemistry. Simply put, functional electron transport chains in intact chloroplasts and mitochondria usually transfer four electrons, from water to reducing equivalents in photosynthesis, and from the citrate cycle to oxygen in respiration. Accidental one-electron transfers can generate ROS that are generally hazardous to the cell. Hydroxyl radical, ^•^OH, can oxidize every molecule, even polymers, usually within 1 ns (Møller et al. 2007). To keep the damage minimal, an extensive antioxidant-protective system has evolved that comprises enzymes (e.g., superoxide dismutase and catalase) and low-molecular-weight metabolites (e.g., ascorbic acid and glutathione; Foyer and Noctor 2005; Halliwell and Gutteridge 2007; Gill and Tuteja 2010). Nonetheless, the underlying ontogenetic effects of accidental oxidative stress constraints may be more fundamental than hitherto anticipated.

Oxygen is the fundamental electron acceptor in aerobic respiration (for electron acceptors in anaerobic respiration see Supplementary Fig. 1). Accidental one-electron transfer reaction products, ROS (Fig. 2), however, can trigger disease, aging, and senescence. Oxygenic respiration and photosynthesis (Fig. 2) produce much more ROS compared with anoxygenic respiration and chemosynthesis. The main advantage, despite the occurring risks, is higher efficacy in terms of energy supply and utilization of more easily available substrates, water and molecular oxygen. These are much more common than any other utilized substrates, such as hydrogen, nitrite, methane, hydrogen sulfide, or ferric iron hydroxide, all of which anaerobic organisms depend on (Supplementary Fig. 1). The success of evolving this efficient energy delivery system, however, entails the concomitant higher risk of exposure to the toxic side products of oxygen, ROS. This idiosyncratic situation is called the oxygen paradox (Davies 1995), and represents a fundamental theme of aerobic life (Halliwell 2006).

Conversely, in low concentrations, ROS have been found to possess beneficial effects. For instance, they trigger cell and tissue differentiation and, moreover, specific enzymes, NADPH oxidases, have evolved that catalyze their formation (Yanes et al. 2010). NADPH oxidases occur in animals (Costantini 2010), plants (Halliwell 2006; Mittler 2017), and fungi (Aguirre et al. 2005). Evolution has found ways to utilize these toxic oxygen by-products for signaling in multicellular organisms (Gomez-Toribio et al. 2009). For this reason, ROS are not completely quenched but rather kept in a homeodynamic balance.

Efforts to maintain certain redox homeodynamics, quenching of accidental ROS with concomitant specific production of ROS signals, e.g., during tissue differentiation, can help to understand why terrestrial plants evolved as sessile organisms. Simply put, the regulatory accommodation of both photosynthesis and aerobic respiration appears to have constrained motility. Otherwise, motile plants would have evolved. Motility presents a further major source of ROS, but also requires neuronal cells for coordination that are extremely sensitive to oxidative damage (Wang and Michaelis 2010). The following insights aim to illustrate the substantial differences in terms of oxidative stress to which plant and animal cells are exposed to. For instance, H_2_O_2_ levels in chloroplasts and peroxisomes in plant cells are estimated to be 30–100 times higher than in mitochondria (Hossain et al. 2015). Hydrogen peroxide can rise from 0.03–1 to 1–100 µMol in stressed plant tissues (Demidchik 2015), whereas in animal mitochondria, the reported H_2_O_2_ only varies between 1 and 100 nMol (Halliwell and Gutteridge 2007). H_2_O_2_ by itself is not a radical, and it is not as hazardous as ^•^OH, but it can be easily converted into the extremely reactive ^•^OH by a single electron transfer (Fig. 2). Its half-life in the cytosol is estimated to be about 1 ms (Møller et al. 2007), which facilitates detection rather more than the much shorter-lived ROS radicals (1 µs–1 ns). The conversion of H_2_O_2_ to ^•^OH is facilitated by transition metals, such as iron and copper, as efficient catalysts of such one-electron transfers (Fenton reaction, Fenton 1894). The presence and coordination chemistry of transition metals can affect ROS formation intensities fundamentally (Edreva 2005; Demidchik 2015; Hadacek and Bachmann 2015). The nucleolus itself is an iron hotspot in plant cells, which is not the case in animal cells (Roschzttardtz et al. 2011). Most of the iron in plants, 80–90%, however, occurs in chloroplasts (Solti et al. 2012).

Additional physiological features underpin the higher level of ROS stress in plant cells. All eukaryotes possess the enzyme catalase (CAT) that converts two molecules of H_2_O_2_ into two molecules of H_2_O and one molecule of O_2_ in peroxisomes. Plants express an additional cell wall-associated enzyme, ascorbate peroxidase (APX), to manage control of H_2_O_2_ concentrations. Especially in peroxisomes, H_2_O_2_ can arise not only from β-oxidation, but also from photorespiration (Gill and Tuteja 2010; Corpas 2015). Elevated temperatures specifically trigger photorespiration in plants. In this process, ribulose-1,5-bisphosphate carboxylase/oxygenase (Rubisco) accepts O_2_ as substrate instead of CO_2_, because higher levels of O_2_ arise from the accelerated photosynthesis. Usually, cytosolic oxygen levels are lower than CO_2_ concentrations. If Rubisco uses O_2_ as substrate, then the formed glycolate is oxidized to glyoxylate in peroxisomes, and the two available electrons are used to reduce O_2_ to H_2_O_2_, which the enzymes catalase and APX destroy by reducing it further to H_2_O (Foyer and Noctor 2009). Rubisco carboxylation evolved early in photosynthetic organisms, before the oxygenation of the atmosphere, when oxygen levels were too low for photorespiration (Foyer et al. 2009). Quenching photorespiration, however, did not improve the efficiency of the Calvin–Benson cycle to produce carbohydrates (Foyer et al. 2009). Quite to the contrary, photorespiration helped to run the Calvin–Benson cycle efficiently in the presence of increasing oxygen concentrations that result from higher photosynthetic rates (Foyer et al. 2009; Timm et al. 2016). The quenching of O_2_ concentrations, the most important precursor of ROS, may thus represent an essential albeit less focused protective metabolic mechanism in aerobic organisms. Likewise, mitochondria provide a similar contribution by decreasing O_2_ concentrations by aerobic respiration (Gross and Bhattacharya 2010). Consequently, higher risks of oxidative stress threaten plant cells more than animal cells that lack chloroplasts. Another ROS that may arise in chloroplasts is singlet oxygen that forms upon energy transfer from excited chlorophyll to the usually present triplet oxygen (Fig. 2). This non-radical ROS undergoes similar reduction reactions to ROS as triplet O_2_, but requires much less activation energy (Krieger-Liszkay 2005). Reactive oxygen species may be involved in triggering cell death in plant cells (Reape and McCabe 2010). The most troublesome characteristic of ROS is their high reactivity and, accordingly, their short half-lives (Demidchik 2015). Damage to cell structures, mostly organic polymers and lipids, is detectable. In vitro experiments also document that ROS can damage DNA by reacting with sugar and base moieties (Cooke et al. 2003).

Land plants have evolved two major adaptations to cope with oxidative stress: first, strong cell walls and vacuoles, and second, production of secondary metabolites acting as efficient antioxidants. The reinforced plant cell wall represents one of the most notable differences between plant and animal cells. Lignified tissues of woody plants mark the extreme and, concomitantly, constitute an important structural component. The conquest of terrestrial habitats has confronted plants with a fundamental physiological constraint to develop control mechanisms of oxidative stress in their attempts to adapt to the harsh terrestrial environmental conditions (Delaux et al. 2012). Complexly structured cell walls may have arisen to help plant tissues withstand draught, daily temperature fluctuations, and high light conditions, as major sources of oxidative stress (Foyer et al. 2009). Cell walls also provide protection against microbial attack (Sarkar et al. 2009). Plants were under pressure not to give up their most valuable physiological trait, photosynthesis, to which water deficit and desiccation can be harmful in causing considerable oxidative stress (Smirnoff 1993).

Autotrophic life entails a rich production of metabolites. Besides the cell wall and chloroplasts, the third characteristic cell organelle of autotrophic plants is the vacuole, usually a large central one. A prominent function of vacuoles is storage of various plant metabolites, primary, secondary, and proteins. Lytic vacuoles, similar to lysosomes in animal cells, exist that take over degradation of various cellular metabolites and organelles (Evert et al. 2009). The central vacuole can take up to 90% of the cell’s volume, which also results in a higher cytoplasmic compartmentalization of cell regions that contain ROS-producing organelles, such as chloroplasts, mitochondria, and peroxisomes, and other less-affected regions that contain the nucleus and the endoplasmic reticulum.

Overall, plants lost motility, and rather optimized an autotrophic, sessile life form during land plant evolution (Supplementary electronic materials, S2). Even “carnivorous” pitcher plants are no exception, as they still have green, photosynthetically active leaves for autotrophy, and they use digestion of insects mainly to improve nitrogen supply on nutrient-poor soils. Pitcher plants have neither evolved motility nor neuromuscular systems, although this would be quite beneficial for catching their prey.

Large vacuoles and other storage cells together with multicellular compartments, such as oil ducts, lactifers, and resin channels, allow plants to accumulate huge amounts of secondary, or as they are more recently called, specialized metabolites (Weng et al. 2012). They share this trait with sessile marine sponges, ascidians, and corals (Proksch 1994; Paul and Puglisi 2004) though many of them are produced by symbiotic bacteria (Piel 2004). The widely occurring flavonoids, for example, are already produced by green algae, an early diverging group in plant phylogeny (Wink 2008). These compounds represent recognized antioxidants that can protect photosynthetically active tissues against accidental oxidative stress, but can also have important roles in plant physiology and development (Buer et al. 2010; Hadacek et al. 2011; Weng et al. 2012; Bartwal et al. 2013; Di Ferdinando et al. 2014). Their widespread occurrence in extant plants underpins the importance of maintaining redox homeodynamics in photosynthetically active cells. The majority of secondary metabolites, however, notably alkaloids, are still viewed predominantly as chemical weapons against microbial pathogens, animal predators, and even competing individuals (Mithofer and Boland 2012). Most of them appeared much later in the phylogeny of land plants, the first time in lycopods (Wink 2008). In context with sexual reproduction, two studies that also focus on secondary metabolites merit mentioning. Both suggest that changes in secondary metabolite composition correlate with the formation of sexual ovules in facultative apomictic plants (Schmidt et al. 2014; Klatt et al. 2016). The observed changes in the secondary metabolite chemistry are not fully understood yet. The present knowledge of potential functions ranges from those that are beneficial to the producer to those that are toxic to predators (e.g., Hadacek 2002). The complex redox chemistry in which most of the known secondary metabolites can be involved just on the basis of their structural properties, allows for various functions, depending on the chemical environment in the compartment in which the metabolite actually is. Moreover, many secondary metabolites can enter coordination complexes with metals that are important cofactors for enzymes and affect metal availability either positively or negatively (Hadacek et al. 2011; Chobot et al. 2014; Hadacek and Bachmann 2015). The inherent danger of a chemistry involving oxygen, the previously pointed-out oxygen paradox of simultaneous efficiency and potential toxicity, also and especially to DNA, requires mechanisms such as sex as a DNA repair system for successful reproduction over many generations. The need for DNA restoration can explain that about 99% of seed plant- and 90% of fern species are obligately sexual (Burt 2000).

To summarize, plants have various adaptations to cope with oxidative stress, namely cell walls, vacuoles on the cells, and antioxidant secondary metabolites. However, these constraints enforce a sessile lifestyle because motility would add another source of oxidative stress that would overburden the cellular regulatory mechanisms.

#### Effects of sessility on distribution of sexes

The physiological constraints of sessile versus motile life forms most likely represented early determinants for hermaphroditic or unisexual sex differentiation in adult organisms (Darwin 1876). For a sessile organism, outcrossing becomes problematic because gametes have to overcome spatial distances by means of external vectors (Fig. 1). Hence, because of their sessile life form, it is important for plants to maximize the number of successful male gamete transfers. This selective pressure was already recognized by Charles Darwin (Darwin 1876). Differences in mobility and mate-search efficiency clearly drove the evolution of separate sexes in animals, and combined sexes in plants and fungi (Eppley and Jesson 2008). Within land plants, a great diversity of sexual systems exists, but combined sexes on one individual, i.e., functional hermaphrodites, are by far most common (Richards 1997). Land plants alternate two generations, the diplontic, differentiated sporophyte (producing meiotically reduced spores), and the haplontic gametophyte (producing gametes). Both generations are primarily sessile. Male gametes are flagellate or ciliate in some early groups (mosses, ferns, Cycads, and *Ginkgo*), and these spermatozoids can actively swim in a liquid medium (e.g., droplets of water, water films, or water inside the ovule). The dependency of fertilization on water is a disadvantage for these early land plant lineages. In conifers and flowering plants, pollen evolved further as a carrier of male gametophytes and gametes. After pollination, the growing pollen tube transports sperm nuclei to the egg cells. Motile stages have been lost in the life cycles of these seed plants (see details in Supplement 2). Passive movement overcomes water dependency and reduces ROS damage in gametes (see electronic Supplementary materials for further details, S2).

Pollen is a passively wind- or animal-transported carrier of the male gametophyte and gametes. This system is quite efficient in overcoming spatial distance between the sessile mating partners, but is inefficient for adult mate search (Eppley and Jesson 2008). Adult mate search is difficult in plants for several reasons. First, passive gamete transport is prone to error for conspecific mating. In flowering plants, less than 1% of the pollen is actually transported to conspecific receptors (Mitchell et al. 2009), even in insect-pollinated plants. The huge loss is due to several factors, such as competing plant species, passive loss during transport, moving of pollen to corbiculae, and feeding by pollen predators, among others (Mitchell et al. 2009). Second, high dependency on high spatial crowding and temporal synchrony hampers successful outcrossing within species (Shuster 2009). Third, the absence of brains and sensory organs limits the ability to perceive compatibility and quality of mating individuals, and hence reduces the efficiency of sexual selection. Even if the plant produces conspicuous floral displays, they rather increase the chances of multiple pollinations from several interspecific pollen donors (Bernasconi et al. 2004) or selfing between flowers of the same individual. These constraints restrict mechanisms of sexual selection to post-pollination processes (Beekman et al. 2016). In seed plants, the style is the main zone for selection among competing pollen tubes and for female choice (Lora et al. 2016). However, these processes are already restricted to 1% of actually produced pollen (Bernasconi et al. 2004). The uncertainty of pollen transfer strongly reduces the strength of sexual selection (Barrett and Hough 2013). All these disadvantages decrease the number of successful male gamete transfers, and would be even more reduced in a population with separate male and female individuals.

The chance of successful gamete transfer is increased by hermaphroditism, as each individual is able to perform outcrossing at the same time as father and mother, which optimizes pollination success (Mitchell et al. 2009). In hermaphroditic populations, all individuals can produce plant embryos. Furthermore, about 40% of all angiosperm species utilize occasional self-fertilization, which provides reproductive assurance even under conditions of low spatial and temporal crowding (Richards 1997; Shuster 2009). However, self-incompatibility (the inability to form seeds after self-pollination; Richards 1997) enforcing outcrossing is the ancestral trait in angiosperms and selected for (Goldberg et al. 2010). Self-compatibility, by contrast, is derived and evolved multiple times at the tips of the angiosperm phylogeny (Goldberg et al. 2010). In the long term, selfing is disadvantageous because of the continued loss of heterozygosity and the potential generation of inbreeding depression, especially for long-lived plants (Charlesworth and Charlesworth 1987; Richards 1997; Goldberg et al. 2010). Accordingly, selfing was obviously not the main selective force for the evolution of hermaphroditism in plants (otherwise, selfing would be ancestral and would appear in all hermaphroditic species). Selfing could be viewed as a derived sideway of reproduction in the case of pollen limitation. Meiosis is still maintained because of the need for DNA repair (Michod 1995; Mirzaghaderi and Hörandl 2016). In contrast, asexual reproduction via seeds (apomixis) occurs in less than 1% of plant species (Mogie 1992; Burt 2000). Apomixis usually remains facultative, with parallel production of sexual and asexual seeds (Hojsgaard and Hörandl 2019). This developmental flexibility is possible because of the late differentiation of germline precursor cells in the flowering buds of adult plants. Plants do need meiotic sex, but as sessile organisms, they do not need separate sexes.

The main selective advantage for a sessile organism is substantially reducing reproductive investment costs by not producing male individuals; costs are reduced to the production of male organs and gametophytes. Furthermore, plant reproduction does not allow for biparental care. In seed plants, only the mother plant can produce nourishing tissues (endosperm) for the embryo within the seed (in angiosperms, the paternal genome contribution is usually required for proper endosperm development, but only the mother provides nutrients; Vinkenoog et al. 2003). If all individuals in a population can be mothers, investment into the next generation is more efficient. These selective forces made combined sexes (either with hermaphroditic flowers or male and female flowers on the same individual) predominant, and dioecy, separate male and female plants, a rare and derived trait. Dioecy occurs in only about 5–6% of angiosperm species (Renner 2014). Notably, about 31% of dioecious plants are wind-pollinated, a percentage that is much higher than in non-dioecious plants (c. 5–6%) (Renner 2014). In wind-pollinated plants, dioecy does not improve floral displays for specific pollinator attraction, and hence it did not evolve under a selective pressure for sexual selection. Dioecy probably evolved to avoid disadvantageous self-pollination in long-lived trees (Renner 2014). Life history trade-offs in resource allocation between male and female individuals further explain why most dioecious plants are perennials (Dorken and Van Drunen 2018). Most dioecious plants do not even have specific sex chromosomes (only 40 species have sex chromosomes, which represents c. 0.3% of dioecious species, and 0.015% of all angiosperm species; Renner 2014). Instead, male/female phenotypes are controlled via mutations or epigenetic changes that cause sterility of either male or female organs in the original hermaphrodite (Ming et al. 2011). Consistent with the scarcity of sex chromosomes, sexual dimorphisms are in plants in general much less pronounced than in animals, and are not necessarily driven by sexual selection (Barrett and Hough 2013; Dorken and Van Drunen 2018).

Concluding this section, oxidative stress and a physiology adapted to photosynthesis enforce a sessile lifestyle in land plants. Sessility, however, favors combined sexes. Hence, we propose that redox chemistry indirectly determines the sexual system of land plants (Fig. 1).

### Effects of oxidative stress on fungi

#### Evolution, physiology, and lifestyle of fungi

The last common ancestor of the clade comprising animals and fungi (ophistokonts) is assumed to have been an aquatic, unicellular, flagellate organism, probably similar to extant choanoflagellates (Lee et al. 2010). Fungi lost flagellate forms and light-sensory organs when they became terrestrial (Lee et al. 2010). They became sessile, with the exception of the basal chytrids (Lee et al. 2010), and abandoned even flagellate zoospores. Terrestrial fungi explore space in their substrates by means of mycelia, similarly as plants do with their roots, as they both require mineral nutrients (Trewavas 2014). Fungi exist as organic matter decomposers, parasites, or symbionts of plants, and as decomposers and pathogens of animals (Deacon 2010).

Fungi are neither motile nor perform photosynthesis themselves. In lichens, fungi form a symbiosis with photosynthesizing algae (electronic Supplementary materials, S3). The major physiological requirement for fungi is organic substrate, for which growth of mycelia or a parasitic/symbiotic lifestyle is advantageous. In all of their lifestyles, exposure to oxidative stress in addition to aerobic respiration is unavoidable. Those who live as saprophytes use oxidative chemistry to degrade polymers such as cellulose and lignin (Baldrian and Valaskova 2008; Jeon et al. 2012). Oxygen plays an important role in decomposing all the huge amounts of biomass that aerobic photosynthesis and respiration generates. Basically, decomposition comprises the same destructive ROS chemistry that endangers living cells, and is known to facilitate disease development (Halliwell and Gutteridge 2007; Hadacek et al. 2011). ROS chemistry represents the driving force in the decomposition of organic matter, especially of carbon polymers such as cellulose, lignin, chitin, and proteins. Especially, the formation of ^•^OH, hydroxyl radical (Fig. 2), triggers chain reactions that yield smaller organic molecules. A minor portion of these oxidation products comprises small organic acids with potential to be fueled into the citric acid cycle of pro- and eukaryotic decomposer organisms that utilize this chemistry for their own benefit (Hammel et al. 2002; Halliwell and Gutteridge 2007; Gomez-Toribio et al. 2009). Fungi that live as plant pathogens have to cope with the oxidative burst in the host plant tissue during the infection process (Kawano 2003). Plant symbiotic mycorrhizal fungi share the stress effects that act on their host, and can contribute to host fitness by strengthening its antioxidant defense (Schützendubel and Polle 2002). Microbes that occur on the surface of plant leaves and on ripening fruits are exposed to high ROS-producing UV stress before they manage to enter the plant tissue (Speijer 2017). As a possible adaptation, *Saccharomyces cerevisiae* has minimized mitochondrial energy production and hence, endogenous ROS production (Speijer 2017). This trade-off supports observations that a saprophytic lifestyle does not combine with energy-demanding motility.

Fungi have a low degree of cell differentiation (Rokas 2008), but nevertheless various sexual systems. About 20% of all fungal species are estimated to be obligately asexual, or they can shift between sexual and asexual life cycles (Burt 2000). Their sexual cycles are mainly designed to reinstall after syngamy diploid chromosome sets that are more robust against oxidative damage and mutation (Lee et al. 2010). Oxidative stress treatments in fission yeast induced sex and increased sexual sporulation rates by 4–18-fold (Bernstein and Bernstein 2013). To summarize, fungi are exposed to various sources of oxidative stress additional to respiration, which enforces a sessile lifestyle to successfully maintain redox homeodynamics.

#### Effects of sessility on sex distribution in fungi

Like plants, fungi as sessile organisms face the problem of bringing mating cells together and require spatial crowding (Fig. 1). However, unlike plants, fungi did not evolve structures that would carry male gametes by external vectors to overcome the distances between mating partners. Gametes are cells of the growing haploid hyphae. Hence, the major challenge for fungi is to increase the chance of any compatible, conspecific mating. Sexual fungi have different mating types encoded by alleles of the MAT locus (Lee et al. 2010). Two or more compatible, different mating types recognize each other by pheromones and fuse cells, and zygotes develop into dikaryotic or syncytial hyphae (Lee et al. 2010). Although a (male) donor function and a (female) recipient function can be discriminated, all individuals can do both. Fungi are thus functionally hermaphroditic (Nieuwenhuis and Aanen 2012). Only after karyogamy, diploid nuclei arise. After meiosis, haploid spores are produced, which grow to form mycelia. Nevertheless, each “individual” mycelium retains the potential for fusion and production of dikaryotic or syncytial hyphae. In populations of ascomycetes, like *S. cerevisiae*, cells can be either homothallic and self-fertile or heterothallic and self-sterile. Ascomycetes reproduce by occasional inbreeding by switches allowing for mating of mother and daughter cells. In basidiomycetes, dikaryotic stages persist until shortly before meiosis and spore formation (Lee et al. 2010). Mating types are determined by alleles of two genes (A, B), resulting in four mating types (tetrapolar systems). However, some species like *Schizophyllum commune* have about 300 A alleles and 90 B alleles, resulting in potentially 27,000 mating types or “sexes”. Such systems are regarded as derived in fungi and occur just in basidiomycetes (Heitman 2015). Hence, basidiomycetes have developed a system that maximizes the chances for outcrossing because all mating combinations are compatible. Theory predicts that in isogamous organisms, mating kinetics will drive the evolution of different mating types, because a new rare mating type will find a new mating partner faster than a common mating type (Iwasa and Sasaki 1987). However, the number of mating types is also influenced by the degree of facultative sexuality: if sex is rare, which is common in isogamous species, then the number of mating types will remain low (Constable and Kokko 2018).

Asexual reproduction is quite common in fungi (Burt 2000; Lee et al. 2010), and they lack pronounced cell and tissue differentiation. Hence, advantages of sexes, such as visual or acoustic mate recognition systems, sexual selection, sex dimorphisms, and divergent sex roles, could not evolve, and fungi cannot exploit the advantages of having males like animals (see section “Oxidative stress imposed on animals”). Mate recognition is limited to pheromone signals and chemotaxis (Lee et al. 2010). In basidiomycetes, a mating preference evolved in dikaryon–monokaryon matings, indicating the presence of sexual selection (Nieuwenhuis et al. 2011). However, it is still unclear on which trait sexual selection is acting (Nieuwenhuis et al. 2011). Altogether, both land plants and terrestrial fungi, but also sessile marine invertebrates (Carlon 1999; Hughes 2005), have a quantity-optimized sexual system in which all individuals of a population can have successful matings and produce offspring (Fig. 1). Sessility, which is enforced by the amount of oxidative stress exposure of fungi, requires such sexual systems.

### Oxidative stress imposed on animals

#### Evolution, physiology, and lifestyle of animals

The oldest metazoan fossils date back to c. 570 Mill. years, and the oldest fossils of bilaterian animal locomotion are c. 565 Mill. years old (Knoll and Nowak 2017). Animals as heterotrophic organisms optimized motility, especially after the full colonization of land in the Silurian (Knoll and Nowak 2017). Motility on land is more demanding than in water. It lacks the buoyant force of water-balancing gravity, and terrestrial landscapes are more structured than aquatic ones. Hence, motile organisms require in general stable (inner or outer) skeletons, muscles, and lots of energy from mitochondria (ATP). Motility of heterotrophic organisms is extremely intensive—quite often, it means running, flying, swimming, or fighting as efficiently as possible. This, however, increases oxidative stress with impairing consequences on muscle contraction in many organisms (Reid 2001; Powers and Jackson 2008).

Coordination of muscle motility requires neuronal systems, and is in most animals facilitated by brains and sensory organs. These organs may confer physiological constraints that are not apparent in direct assessments of potential stress factors. Neuronal systems are highly sensitive to oxidative stress, and ROS formation is involved in many neurodegenerative diseases (Wang and Michaelis 2010; Speijer 2011; Yan et al. 2013; Cobb and Cole 2015; Raina and Sen 2018). The effects of ROS on neurons depend on concentrations and types of neuronal cells (Wang and Michaelis 2010). For instance, while low concentrations can have positive signaling effects, high concentrations can attenuate long-term potentiation and synaptic transmission (Wang and Michaelis 2010). Notably, brain cells are most prone to oxidative damage because of their high oxygen demand, the presence of catalytic metals (iron and copper) for the radical-producing Fenton reaction, by the presence of polyunsaturated fatty acids, and by their low regeneration capacity as post-mitotic cells (Wang and Michaelis 2010). Somatic DNA repair mechanisms of oxidative damage are potentially mutagenic that causes neurological problems (Li et al. 2018).

Perhaps, the sensitivity of brains against oxidative damage and mutagenesis is a major reason for the need of sex as a regular DNA restoration mechanism in animals. This DNA restoration comprises homologous recombinational DNA repair during meiosis and the selective elimination of mutations in the germline (Hörandl 2009). Hence, the zygote can start development with healthy genes for all organs (Hörandl 2009). This DNA restoration is more important for neuronal cells than for other energy-consuming cell types, because neurons cannot be functionally replaced after damage-induced cell death (Speijer 2017). In support of this hypothesis, birds and mammals, with their highly developed brains, do have obligate sex only, while otherwise asexuality is scattered and widespread in the metazoan phylogeny (Simon et al. 2003). More than 99% of metazoan species are obligately sexual (Burt 2000); few examples of ancient asexuals are marine or aquatic (Butlin 2002), where motility is less stressful (see the beginning of this section). Bdelloid rotifers, a prominent example for an ancient asexual animal living in ponds, showed an extraordinary high resistance against oxidative stress during exsiccation, and a highly efficient DNA repair system (Hecox-Lea and Mark Welch 2018). They probably use gene conversion for elimination of deleterious mutations (Flot et al. 2013).

The combination of motility with another major source of stress (e.g., photosynthesis or decomposition) is apparently only possible in special symbioses. Notably, the only motile organism performing a symbiotic photosynthesis is a marine one, the sacoglossan mollusk *Elysia* (see Supplementary electronic materials, S3). Strikingly, no terrestrial organism exists that combines motility using neuromuscular systems and photosynthetic autotrophy. Similar constraints of redox homeodynamics require that animals are not decomposers, but use the help of endosymbiotic bacterial communities (gut microflora) to be able to digest large organic biomolecules like lignin and cellulose (e.g., ruminant animals or termites).

Mammals do not synthesize and accumulate comparably conspicuous amounts of secondary metabolites as plants do. Quite on the contrary, they rely mostly on the acquisition of many vitamins from plant food sources, many of which are structurally similar to secondary metabolites and hormones (Hadacek and Bachmann 2015). Instead, a highly developed immune system takes over from secondary metabolites as defense against microbial pathogens. In the framework of this system, phagocytes, such as neutrophils and monocytes, attack parasites by ROS production during engulfment attempts (El-Benna et al. 2005).

Animals comprise more different cell types than sessile eukaryotes, including neuromuscular systems. This indicates a strong evolutionary process (Rokas 2008), against which the concurrent establishment of photosynthesis might have acted as a too strong constraint. Notably, brains cannot use fatty acids as a “fuel”, because β-oxidation of fatty acids causes severe oxidative stress to which neuronal cells are highly sensitive. Whereas short-chained fatty acid β-oxidation occurs within mitochondria, to provide direct substrate availability for ATP synthesis need for motility, peroxisomes take over the breakdown of long-chain fatty acids (>22C atoms). Sessile plants, by contrast, perform their β-oxidation in peroxisomes exclusively, because ATP is also provided by the chloroplast and none is required for motility (Poirier et al. 2006; Speijer 2011; Schönfeld and Reiser 2013; Speijer 2017). Fungi do the same, because they are not motile. These metabolic characteristics have emerged to, in concert with other traits, facilitate systems that either accommodate photosynthesis or allow for motility. More energy equivalents are required for motility in animals and, consequently, less are available for secondary metabolite biosynthesis. The specific compartmentation of metabolic pathways together with the absence of photosynthesis appears not to require an additional antioxidant shield in terrestrial ecosystems that secondary metabolites would provide (Johnson 2003).

#### Effects of motility on sexes in animals

Features related to motility (skeleton, eyes) and dioecy belong to the most important factors for the diversification of animals (Jezkova and Wiens 2017). Motility is a big advantage for outcrossing sex, as individuals can actively move to the mating partner, which makes sex possible over a larger spatial distance and independent from external vectors (Fig. 1). Copulation with direct physical contact is widespread among terrestrial animals, and it ensures precise placement of the male gametes without much loss before mating. Highly developed sensory organs and brains that mobility requires concomitantly improve active mate perception, choice, and compatibility (Eppley and Jesson 2008). Mating behavior evolved to help recognizing not only a conspecific partner but also the best mating partner among the conspecific individuals, which has the advantage of sexual selection (Shuster 2009). The principle of sexual selection was already recognized by Darwin (1859): with differentiation of sexes, sexual selection can act mostly before copulation as male–male competition, or as female choice. We cannot provide a comprehensive review on this broad topic here, but we just want to highlight some relevant points. A meta-analysis on 66 species over the animal kingdom confirmed the Darwin–Bateman paradigm (Janicke et al. 2016) that males are under stronger sexual selection than females, and that sexual selection drives sexual dimorphisms and divergent sex roles (Janicke et al. 2016). Sexual selection can act most efficiently with two different sexes: it increases the efficacy of selection against deleterious mutations as males are under a stronger selection than females (Agrawal 2001; Lumley et al. 2015). Sexual selection can explain the evolution of obligate sexuality in diploid bisexual systems, which is due to the high fertilizing success of fit males (Kleiman and Hadany 2015). With separate sexes, division-of-labor advantages of sexes can be effective in various ways: for instance, biparental care for feeding juveniles exists in many vertebrate groups, and synergistic effects select for task specialization (Barta et al. 2014). Different parental investment and sexual selection reinforce divergent sex roles (Fromhage and Jennions 2016; Janicke et al. 2016). Recent studies on birds suggest that sexual selection influences the amount of biparental care (Liker et al. 2015). Taken together, existing theoretical and empirical studies support the hypothesis that separate sexes confer evolutionary benefits.

In hermaphroditic animals, sexual selection is halved compared with that of population with pure males (Greeff and Michiels 1999). This disadvantage explains that hermaphroditism occurs only in 5% of the animal species (Jarne and Auld 2006). Notably, even hermaphroditic motile animals usually do not necessarily self-fertilize (Beukeboom and Vrijenhoek 1998; Jarne and Auld 2006). Similarly to plants, selfing in animals can be viewed just a side-strategy for maintaining reproductive assurance under specific ecological conditions with mate limitation (Jarne and Auld 2006). However, the low actual frequency of selfing in animals compared with plants supports the hypothesis that motile organisms need such a side-strategy much less than sessile organisms.

The genetic control of sex determination is in animals strongly established via sex chromosomes. The MAT locus of the opisthokont ancestor evolved further into various animal sex determination systems (Lee et al. 2010), suggesting that differentiation of sexes in the animal kingdom is an ancestral, genetically determined feature. Phylogenetic reconstructions could not clearly resolve whether combined or separate sexes represent the ancestral state (Sasson and Ryan 2017). This uncertainty is mainly due to alternative tree topologies, and to the fact that many basal animal groups are marine and hermaphroditic. In animals with separate sexes, only about 50% of individuals, the females, can produce offspring—but with a higher certainty of reproductive success and quality of offspring because of motility, sensory organs, and mating behavior allowing for sexual selection. Altogether, animals require a quality-oriented sexual system to meet the physiological constraints of motility, and they optimize it by differentiation of two sexes (Fig. 1).

### Conclusions and outlook

We propose that physiological constraints, such as maintenance of redox homeodynamics, might have contributed fundamentally to shaping of the evolution of sexes, albeit in indirect ways. Oxidative stress constrains sessile and motile lifestyles, because the three main metabolic systems (photosynthetic/heterotrophic/saprotrophic) cannot coexist within one organism, especially in terrestrial biota. We propose a trade-off mechanism that eukaryotic organisms can balance redox homeodynamics only for two ROS-producing metabolic systems (respiration plus photosynthesis, or respiration plus decomposition, or respiration plus motility). The adaptations required for these metabolism combinations preclude the establishment of the third source of ROS, respectively. The few exceptions from this rule all rely on symbioses (Supplementary electronic material S3). In turn, lifestyles and physiology determine the evolution of sexes indirectly, because sessile organisms (plants and fungi) lack efficient mate-search systems and require quantity-optimizing reproductive systems (hermaphroditism and homothallism). In contrast, motile organisms (most animals) need quality-optimizing systems (separate sexes with females and males) and can benefit best from sexual selection (Fig. 1).

To test these hypotheses, multidisciplinary approaches are needed. Basic physiology and redox homeodynamics in eukaryotic cells are usually just studied in a few model organisms, while the vast majority of species have not yet been analyzed. It would be interesting to compare sessile/mobile and aquatic/terrestrial species to test the hypothesis that terrestrial life requires more specific adaptations to cope with oxidative stress. The advantages of sexual systems, of sex ratios, and various scenarios of sexual selection need to be rigorously screened and evaluated statistically to compare sessile versus mobile lifestyles. Here the comparison of contrasting systems in animals, plants, and fungi will be more informative than just analyses within these kingdoms. We anticipate that such a broad view on all eukaryotic multicellular organisms could result in a unifying theory, and resolve the paradox of the “cost of males” as an adaptation to motile lifestyle.

## Supplementary information

Supplement 1. Prokaryotic anaerobic chemosynthesis and respiration

Supplement 2. The life cycle of land plants and selective pressures for hermaphroditism

Supplement 3. Organisms with symbiontic photosynthesis: a) Lichenes and b) photosynthetic slugs
