# Supplementary material for: Oxygen, life forms, and the evolution of sexes in multicellular eukaryotes
Source: Heredity (Edinb). 2020 May 15;125(1-2):1–14. doi: 10.1038/s41437-020-0317-9 (PMC7413252; doi:10.1038/s41437-020-0317-9)
Supplement: Supplementary file 1 — Supplement 1. Prokaryotic anaerobic chemosynthesis and respiration [file 41437_2020_317_MOESM1_ESM.pdf]

Supplementary electronic materials S1:

Prokaryotic anaerobic chemosynthesis and respiration

Electron donors

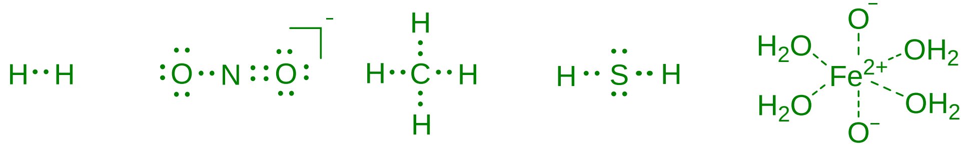

H_2	NO_2^-	CH_4	H_2S	$Fe(II)OH)_2$
Hydrogen	*Nitrite*	*Methane*	*Hydrogen sulphide*	*Ferric iron hydroxide*

Electron acceptors

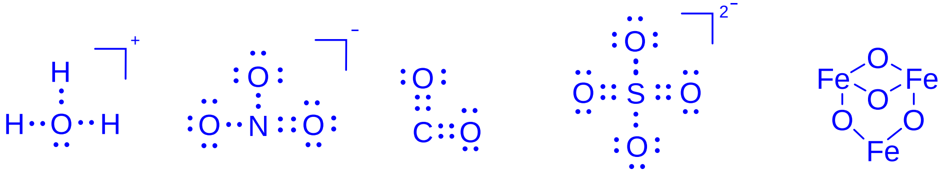

H_3O^+	NO_3^-	CO_2	SO_4^{2-}	Fe_3O_4
Hydronium ion	*Nitrate*	*Carbon dioxide*	*Sulphate*	*Iron oxide*

Arkhipova OV, Akimenko VK (2005) Unsaturated organic acids as terminal electron acceptors for reductase chains of anaerobic bacteria. *Microbiology* **74**:629-639.

Grein F, Ramos AR, Venceslau SS, Pereira IAC (2013) Unifying concepts in anaerobic respiration: Insights from dissimilatory sulfur metabolism. *Biochimica Et Biophysica Acta-Bioenergetics* **1827**:145-160.

Richardson DJ, Berks BC, Russell DA, Spiro S, Taylor CJ (2001) Functional, biochemical and genetic diversity of prokaryotic nitrate reductases. *Cell Mol Life Sci* **58**:165-178.

Richter K, Schicklberger M, Gescher J (2012) Dissimilatory reduction of extracellular electron acceptors in anaerobic respiration. *Applied and Environmental Microbiology* **78**:913-921.
