# Supplementary material for: Oxygen, life forms, and the evolution of sexes in multicellular eukaryotes
Source: Heredity (Edinb). 2020 May 15;125(1-2):1–14. doi: 10.1038/s41437-020-0317-9 (PMC7413252; doi:10.1038/s41437-020-0317-9)
Supplement: Supplementary file 2 — Supplement 2. The life cycle of land plants and selective pressures for hermaphroditism [file 41437_2020_317_MOESM2_ESM.pdf]

Supplementary electronic materials S2:

The life cycle of land plants and selective pressures for hermaphroditism

Land plants alternate two generations, the diplontic sporophyte (producing meiotically reduced spores) and the haplontic gametophyte (producing gametes). Both generations are primarily sessile, only male gametes have in some groups an active mobility. In bryophytes, the gametophyte is the green, metabolically active, long-lived generation; in most lycophytes and ferns, the gametophyte is successively reduced, but mostly still a photosynthetically active plantlet, whereas the sporophyte becomes the big, long-lived and differentiated green plant. In seed plants, the sporophyte is the only photosynthetically active, differentiated generation, while the gametophytes become small, organ-like structures (pollen).

In mosses and liverworts, lycopods and ferns, sprinkling raindrops or water streamlets distribute flagellate, mobile male gametes from one gametophyte to the other. After arrival on the female organs, they swim actively towards the egg cell inside the archegonium. Spatial crowding of individuals and on wet habitats enhance fertilisation success. Hence, asexual pathways, vegetative propagation, or self-fertilization (= selfing), represent common alternatives to outcrossing (Mogie 1992). However, these modes of reproduction are biased by a loss of genetic diversity. Self-fertilization can happen in mosses easily, either within the same monoicous gametophyte (intragametophytic selfing) or between spatially near dioicous gametophytes (intergametophytic selfing) that have resulted from spores from the same meiosis. Both selfing modes result in a rapid loss of heterozygosity within populations (Eppley et al. 2007). Similar constraints act among the small, sessile gametophytes of ferns.

In seed plants, the gametophytes are reduced to few-celled, organ-like structures that are no longer self-sustaining organisms. Pollen evolved as a non-motile, passively wind or animal-transported carrier of the small male gametophyte (Rudall and Bateman 2007), while the female gametophyte develops completely within the sporophyte. The sporophyte, the green plant, is mostly hermaphroditic, ie. it produces both male and female gametophytes, either within the same flower, or in different flowers of the same plant (monoecy) (Richards 1997). Various combinations exist in angiosperms, but individuals carrying male or female gametophytes only (dioecy), as comparable to males and females in animals, occur only in 5-6% of species (Richards 1997, Renner 2014). Hence, most angiosperms can be regarded as functionally hermaphroditic.

Seed plant evolution is characterized by a successive independence of water for fertilization. In gymnosperms just a pollination droplet produced by the plant is needed to suck in the pollen grain, and the primitive lineages (cycads) still have mobile male gametes to swim within the archegonial chamber towards the egg cell. In conifers and in angiosperms, the pollen tube transports male, immobile sperm nuclei towards the micropyle and releases them there for fertilization (siphonogamy). The light-weighted pollen can be transported over much longer distances from one individual to the other by wind or by animals, and fertilization is independent from external water.

Concomitantly, land plant evolution is characterized by a successive dominance of the sporophyte as the photosynthetic and metabolically active, biomass-producing generation. Notably, leaves, the photosynthetic organs, are continuously renewed, even in evergreen plants, because degradation of

chloroplast DNA during photosynthesis limits their life span (Oldenburg and Bendich 2015). In seed plants, the female gametophyte bearing the egg cell is deeply embedded in tissues and keeps plastids inactive and protected from ROS. Plastids are mostly inherited via egg cells. In the male gametophyte development, plastids are originally transmitted to the sperm cells, but they either degrade until maturity of the male gamete (Primavesi et al. 2017), or degradation of organelles of one parent in the zygote leads to uniparental organelle inheritance (Zhang and Zheng 2016).

Hence seed plants keep the bisexual system for gametophytes only to benefit from uniparental organelle inheritance and the division of labour principle: male gametes bridge spatial gaps between mating partners, and immobile, inactive female gametes transmit mitochondria and plastids (see main text). Plants minimize ROS-induced damage from photosynthesis and respiration in their gametes. But, the sporophyte – the green plant – is mostly functionally hermaphroditic as female and male gametophytes and gametes, respectively, are produced on the same plant (includes also monoecy). This distribution of sexes is best explained by the fact that a sessile organism has to maximize the amount of successful pollinations, simply by having all individuals acting both as mother and as father.

Eppley SM, Taylor PT, Jesson LK (2007) Self-fertilization in mosses: a comparison of heterozygote deficiency between species with combined versus separate sexes. Heredity 98:38–44.

Mogie M (1992) The evolution of asexual reproduction in plants. Chapman & Hall, London.

Oldenburg DJ, Bendich AJ (2015) DNA maintenance in plastids and mitochondria of plants. Front Plant Sci 6:883.

Primavesi LF, Wu HX, Mudd EA, Day A, Jones HD (2017) Visualisation of plastid degradation in sperm cells of wheat pollen. Protoplasma 254:229–237.

Renner SS (2014) The relative and absolute frequencies of angiosperm sexual systems: dioecy, monoecy, gynodioecy, and an updated online database. Am J Bot 101:1588–1596.

Richards JA (1997) Plant breeding systems. Chapman and Hall, London.

Rudall PJ, Bateman RM (2007) Developmental bases for key innovations in the seed-plant microgametophyte. Trends Plant Sci 12:317–326.

Zhang M, Zheng CX (2016) Archegonium and fertilization in Coniferopsida. Trees-Struct Funct 30:75–86.
