# Supplementary material for: Oxygen, life forms, and the evolution of sexes in multicellular eukaryotes
Source: Heredity (Edinb). 2020 May 15;125(1-2):1–14. doi: 10.1038/s41437-020-0317-9 (PMC7413252; doi:10.1038/s41437-020-0317-9)
Supplement: Supplementary file 3 — Supplement 3. Organisms with symbiontic photosynthesis: a) Lichenes and b) photosynthetic slugs [file 41437_2020_317_MOESM3_ESM.pdf]

Supplementary electronic materials S3:

Organisms with symbiontic photosynthesis: a) Lichenes and b) photosynthetic slugs

a) Lichenes (lichenized fungi):
Lichens represent an alternative form of sessile symbiotic life that has colonized terrestrial habitats. Nearly one-fifth of all known fungal species accommodate algal species in the mycelial tissues. The algal partner provides photosynthetic products, the fungus nutrients. In this symbiosis, the fungal partners have somehow managed to develop "plant-like physiology" and can form a plant-like superorganismal thallus albeit lacking the many cell types and degree of tissue differentiation of the latter (Sanders 2001). Lichens, especially the fungal symbiont partners, are talented producers of secondary metabolites, many of which form aromatic ring systems and have attracted pharmaceutical interest (Muller 2001). Besides of antimicrobial and –herbivore properties, usnic acid, a common lichen secondary metabolite, increased after high light stress (Caviglia et al. 2001), resembling flavonoids and anthocyanins occurring widely in plant leaves. Assumedly, they can add protection against high oxidative stress during photosynthesis (Grace and Logan 2000).

b) Elysia: Some members of Sacoglossa sea slugs can incorporate their algal food functionally intact into their own cytosol (functional kleptoplasty). The marine sarcoglossan mollusc Elysia chlorotica feeds on the green algae Vaucheria chlorotica. After digesting, the algal chloroplasts are retained within the cytoplasm of cells that line the digestive tract. If only light and air is provided, photosynthesis by the captured plastids (kleptoplasts) provides the sea slug with energy for duration of its whole life-span (9–10 months in average). No horizontal gene transfer of algal genes into the sea slug's nucleus could be documented so far (Rumpho et al. 2011, Bhattacharya et al. 2013). It is not known how Elysia species deal with toxic ROS that are produced by the sequestered chloroplast (Rauch et al. 2015), but species-specific ROS tolerance appears to influence the time of their survival (de Vries et al. 2015). However, these rare exceptions of photosynthetic animals evolved only in marine habitats and under conditions of slow motility.

Bhattacharya D, Pelletreau KN, Price DC, Sarver KE, Rumpho ME (2013) Genome analysis of Elysia chlorotica egg DNA provides no evidence for horizontal gene transfer into the germ line of this kleptoplastic mollusc. Mol Biol Evol 30:1843–1852.

Caviglia AM, Nicora P, Giordani P, Brunialti G, Modenesi P (2001) Oxidative stress and usnic acid content in Parmelia caperata and Parmelia soredians (Lichenes). Farmaco 56:379–382.

de Vries J, Woehle C, Christa G, Wagele H, Tielens AGM, Jahns P, Gould SB (2015) Comparison of sister species identifies factors underpinning plastid compatibility in green sea slugs. Proc Biol Sci 282:1802.

Grace SC, Logan BA (2000) Energy dissipation and radical scavenging by the plant phenylpropanoid pathway. Phil Trans R Soc London B-Biol Sci 355:1499–1510.

Muller K (2001) Pharmaceutically relevant metabolites from lichens. Appl Microbiol Biotechnol 56:9–16.

Rauch C, de Vries J, Rommel S, Rose LE, Woehle C, Christa G, Laetz EM, Wagele H, Tielens AGM, Nickelsen J, Schumann T, Jahns P, Gould SB (2015) Why It Is time to look beyond algal genes in photosynthetic slugs. Genome Biol Evol 7:2602–2607.
Rumpho ME, Pelletreau KN, Moustafa A, Bhattacharya D (2011) The making of a photosynthetic animal. J Exper Biol 214:303–311.
Sanders WB (2001) Lichens: The interface between mycology and plant morphology. Bioscience 51:1025–1035.
